# Depression and its associated factors among adult women in Bangladesh during the July 2024 revolution

**DOI:** 10.1007/s44192-026-00470-z

**Published:** 2026-05-25

**Authors:** Shobhan Das, Subash Thapa, Tanmay Mandal, Hemayet Hossain, Fahad Ahmed, Md. Moinul Kabir Bijoy, Sumaya Shargin Khan, Mohammad Nahian Rahman, Tanvir Ahmad, Suchona Akter, Afroza Sultana Nitu, Margia Akter, A. S. M. Mohiuddin, Md. Shahidur Rahman Chowdhury, Bashudeb Paul, Jing Kersey, Md. Mahfujur Rahman, Md. Masudur Rahman

**Affiliations:** 1 Jiann-Ping Hsu College of Public Health, Georgia Southern University, Statesboro, GA 30460 USA; 2 Mymensingh Medical College, Mymensingh, 2200 Bangladesh; 3 Department of Anatomy and Histology, Sylhet Agricultural University, Sylhet, 3100 Bangladesh; 4 Department of Microbiology and Public Health, Gazipur Agricultural University, Gazipur, 1706 Bangladesh; 5 Department of Medicine, Sylhet Agricultural University, Sylhet, 3100 Bangladesh; 6 Department of Pathology, Sylhet Agricultural University, Sylhet, 3100 Bangladesh

**Keywords:** Depression, Political unrest, Women’s mental health, Bangladesh, July 2024 revolution

## Abstract

**Background:**

Political unrest heightens women’s vulnerability to psychosocial distress and depression. The July 2024 revolution in Bangladesh caused major societal disruption, yet its mental health impact on women is poorly understood. This study assessed the prevalence and associated factors of depression among adult women during this period of unrest.

**Methods:**

A cross-sectional design was conducted among 687 adult women using a structured, validated questionnaire. Depression was assessed through a Patient Health Questionnaire-9 (PHQ-9) with seven self-reported health outcomes. A PHQ-9 score ≥ 10 is considered depression; otherwise, no depression. A binomial Logistic regression and a Pearson Chi-square test were used to identify factors associated with depression.

**Results:**

Overall, 54.87% of women reported depressive symptoms, with the highest prevalence among young women aged 18–34 years, unmarried, university-educated, and urban residents. Nervousness (91.70%), loss of pleasure (86.03%), and anxiety (85.15%) were the most frequently reported symptoms. Social media exposure contributed to depressive symptoms in 23.73% of participants. Nervousness symptoms had significantly (*p* < 0.05) higher proportions of depression (52.84%). Women with family members directly affected by unrest had 2.15 times higher odds of depression (AOR: 2.15, 95% CI 1.35–3.40). The self-reported symptoms suffocation (AOR = 2.10, 95% CI 1.33–3.32), loss of pleasure (AOR = 3.59, 95% CI 1.91–6.74), and energy loss (AOR = 1.70, 95% CI 1.13–2.55) were significantly associated with higher odds of depression, *p* < 0.05.

**Conclusion:**

During the July 2024 revolution, Bangladeshi women faced significant psychological distress. Targeted mental health support, monitoring social media impacts, and community counseling in unrest-affected areas are essential, while future research should examine long-term outcomes and effective interventions.

## Introduction

The July 2024 revolution in Bangladesh marked one of the most unstable socio-political movements in the nation’s recent history. Sparked by deep dissatisfaction with governance, widespread corruption, and perceived injustices to the young people in government jobs [1]. The movement was initially spearheaded by students and young activists, and was later joined by teachers, professionals, and ordinary citizens, all demanding accountability and structural reforms in the quota system for government jobs in Bangladesh [2]. As demonstrations intensified, the security forces responded with severe crackdowns, including open gunfire and indiscriminate assaults on unarmed civilians [3].

The July Revolution unfolded between July 16 and August 5, 2024, resulted in a tragic and substantial loss of life. The UN Office of the High Commissioner for Human Rights estimated death of over 1400 people including children and victims of extrajudicial killings [1, 4]. UN OHCHR also reported 12–13% of those killed were children, and many women faced sexual and gender-based violence [1]. A significant number of individuals suffered life-changing disabilities/injuries (22,000 people), including permanent vision loss (92 people) due to rubber bullets and tear gas shell injuries. Children caught in the unrest also lost their lives, further amplifying the grief and trauma of families [5]. The visibility of killings in broad daylight, the suppression of voices, and the brutality of the state response left communities shocked and devastated.

From actively participating in protests to strategizing and coordinating activities, women contributed at every level. Such widespread violence and loss have long-term implications for mental health [6]. Women, in particular, have been disproportionately affected. Witnessing deaths of family members, economic disruptions, and persistent uncertainty about safety have pushed many into psychological distress. Self-reported accounts reveal symptoms such as persistent sadness, anxiety, hopelessness, and loss of interest in daily life, consistent with depression. Social stigma, limited access to mental health care, and lack of support networks further aggravate this burden [1].

Depression is a psychological concept, feeling sad, and thinking negatively [7]. According to the World Health Organization, depression is a mental disorder characterized by low mood and loss of pleasure for a long period of time [8]. Fifteen subtypes of depression are classified based on symptoms, etiology, time of onset, sex, and treatment. Ni et al. (2020) revealed that in the political unrest area, the range of post-traumatic depression level was 4–41% [9]. There are various social determinants, such as emotional, physical torture, sexual abuse, neglect, domestic violence, food insecurity, natural disasters, terrorist acts, and military combat, political unrest, homelessness, incarceration, and migration status, that increase depression [10].

War and political unrest can negatively contribute to mental and physical health among the citizens [11]. Any Violence can affect people negatively, and women in unstable regions often face higher stress and risk of depression [12]. Political unrest undermines women’s emotional, physical, and financial well-being, with unstable regions linked to heightened distress and greater vulnerability to depression [12, 13]. The mental trauma leads to depression, which significantly impacts women’s mental health. Empirical evidence revealed that traumatic experiences often increase the risk of suicide [14, 15].

Jayatunge et al. (2020) revealed that political violence in Sri Lanka causes serious mental health problems, especially for women, leading to depression, anxiety, trauma, loss of trust, and a decline in overall well-being [16]. In 2009, during a period of political unrest in Sri Lanka, the prevalence of anxiety and depression was 32.6% and 22.2% respectively [17]. Polyvianaia et al. (2025) found that the Ukraine war increased depression and suicide risk, with women and students showing especially high levels of depression and anxiety even after 20 months [18]. A meta-analysis revealed that in Syria, the depression level of the refugees was 27–54% [19]. Amiri and Khan (2025) described from national data of Iran that depressive burden has increased from 1990 to 2021 among young adults [20]. Israeli women had heightened vulnerability to depression from the war exposure [21]. A community study in Ethiopia shows substantial depression due to war and siege [22].

According to the World Health Organization (WHO), depression and anxiety cost the global economy US$1 trillion per year in lost productivity; scaling evidence-based care yields a 4:1 return on investment. Across Low- and Middle-Income Countries, mental-health conditions cost 0.5–1.0% of GDP, with low per-capita costs to scale core packages [23]. Despite ample evidence of women’s elevated vulnerability to depression and anxiety in Bangladesh, particularly in rural areas, where up to 26% of women report mental health problems influenced by socioeconomic stressors and poor health access [24]. A few studies have explicitly examined how political unrest**,** such as the 2024 July Revolution, affects depressive symptoms among Bangladeshi women. One hospital-based study found alarmingly high rates of depression (82.5%) among largely male survivors of the uprising [25]. Yet the mental health burden among female survivors or those experiencing unrest without hospitalization remains undocumented. Furthermore, while qualitative evidence highlights women’s restricted mobility and exposure to threats during periods of political turmoil [26], specific linkage between unrest‐related stressors and women’s self-reported depressive symptoms has not been quantitatively assessed. This lack of gender-sensitive, population-level data represents a critical research gap in Bangladesh’s mental health literature.

Although the psychological consequences of political unrest have been documented in war-affected regions such as Syria, Ukraine, and Ethiopia, [27, 28] limited attention has been given to countries like Bangladesh, where political instability and episodes of violence have been recurrent for decades. Most existing research in Bangladesh has focused on economic impacts, social disruption, and public health outcomes, with relatively few studies systematically examining the relationship between political unrest and mental health, particularly depression. Women, who often bear the dual burden of household responsibilities and community vulnerabilities, are disproportionately affected, yet their voices remain underrepresented in empirical research. Moreover, much of the available evidence relies on clinical diagnoses, leaving a gap in understanding the prevalence of self-reported depressive symptoms among women exposed to unrest. Addressing this gap is critical for informing gender-sensitive mental health interventions and policymaking in Bangladesh. Motivated by the limited evidence on women’s mental health in post-revolution contexts, this study aims to examine the prevalence of depression and its associated factors among adult women in Bangladesh following the July 2024 revolution. The objective is to identify key sociodemographic, economic, and psychosocial determinants linked to depressive symptoms. The central research question is: what factors are associated with depression among adult women in this setting? We hypothesize that exposure to social disruption, economic hardship, and reduced social support increases the likelihood of depression. This study is important for informing targeted public health interventions and policies to address women’s mental health needs in post-crisis Bangladesh.

## Materials and methods

### Study design

A cross-sectional study design was conducted among the adult women in Bangladesh. This study was designed as a cross-sectional investigation to assess the prevalence of depression and its associated factors among adult women in Bangladesh during the July 2024 revolution. Data was collected at a single point in time, enabling the examination of relationships between depressive symptoms and relevant sociodemographic, economic, and psychosocial variables. This design is appropriate for identifying patterns and potential risk factors within a population during a specific post-revolution context.

### Study location and duration

The study location was across the eight divisions of Bangladesh. Among the eight divisions, 46 districts were covered during data collection. Data were collected immediately after the ongoing political revolution in Bangladesh in July 2024. The revolution began at the beginning of July 2024 and ended on August 5, 2024. The data collection started on August 14 and ended on September 12, 2024. This timeframe allowed for the recruitment of participants, administration of structured questionnaires, and collection of relevant self-reported health outcomes from adult women across diverse regions of Bangladesh.

### Study population

The study population consisted of adult women aged 18 years and above residing in Bangladesh. The adult women were recruited from both urban and rural settings using a non-probability convenience sampling approach. A total of 687 participants were interviewed during the survey period using a structured, validated questionnaire. Women were recruited from both urban and rural communities to capture diverse sociodemographic and health backgrounds. The inclusion criteria of the participants were that they must be citizens of Bangladesh, must have been following the ongoing July revolution by using various media, and be willing to take part in this study. Women who could communicate effectively and were willing to participate were eligible. Exclusion criteria included those with severe cognitive impairment, serious illness preventing participation, or unwillingness to complete the interview. Pregnant women in critical condition were also excluded to avoid potential bias in reporting health outcomes. The mentally ill women were also excluded from this study.

### Sample size calculation

An unknown population formula was used to calculate sample size. The required participants were determined using the formula: N = $\frac{{\left[ {\left( Z \right)^{2} \times P \times \left( {1 - P} \right)} \right]}}{{\left( d \right)^{2} }}$. To complete this calculation, 95% Confidence Interval (CI), 50% Proportion of the Population (P), and % Margin of error (d) are considered. Therefore, from the formula, the sample size is N = $\frac{{\left[ {\left( {1.96} \right)^{2} \times 0.50 \times \left( {1 - 0.50} \right)} \right]}}{{\left( {0.05} \right)^{2} }}$ = 384.16 ≈ 385. The calculated minimum sample size was therefore approximately 385 participants. To enhance the precision and reliability of the study outcomes, as well as to allow broader generalization, the sample size increased by 78.44%, resulting in a final sample of 687 participants [29].

### Data collection

Data was collected by using a structured, validated Patient Health Questionnaire-9 (PHQ-9). The PHQ-9 was used for depression assessment, and other structured questions were used for identifying the sociodemographic status, depression influencing factors, lifestyle of the participants, and depression-related symptoms. The data were collected by three methods: Google Forms, in-person interviews, and cellphone interviews. The entire study data was collected just immediately after the July 2024 revolution by eleven of our study team members. The data collection was done in the middle of September 2024. In the data collection instruments, there were three sections. The basic and socio-economic information were in Section A and self-reported health outcomes were in Section B. In Section C, there were the main mental stress-related questions (PHQ-9 questionnaire). Finally, in Section D, there were only participants’ habitual fact-related questions. 

#### Depression classification

The Patient Health Questionnaire-9 (PHQ-9) is one of the most widely validated and commonly used screening instruments for depression. It includes nine items, each scored from 0 = Not at all, 1 = Several days, 2 = More than half the days, and 3 = Nearly every day, resulting in a total score ranging from 0 to 27. Based on established cut-offs, scores of  ≥ 10 indicate depression. A score of < 10 is generally considered to be no depressive symptoms [29].

### Health outcomes

After a literature search, we consider the seven most common depression-related health outcomes. Participants were asked about a set of common depression-related symptoms that is associated with psychological distress. These symptoms included anxiety, frequent urination, insomnia, loss of energy, loss of pleasure, nervousness, and sensations of suffocation [30]. Each outcome was self-reported using a structured questionnaire, with dichotomous response options (yes/no). Self-reporting was chosen to capture participants’ subjective experiences, which are particularly relevant in the context of depression research, where somatic and emotional symptoms often overlap [31–33].

### Study variables

During data collection, a total of 23 variables were considered for the data collection. Among these variables, seven were self-reported health outcomes, eleven were demographic and socio-economic factors and five were habitual facts-related. Besides these, a set of nine questions was considered as the PHQ-9. The set of nine questions was used to calculate the mental stress level of the participants. Depression level was determined from the PHQ-9 score, which was used as the dependent variable. However, five habitually fact-related extra questions were added in the questionnaire. During the data analysis, these variables were not considered due to a poor significance level.

### Statistical approach

For the data analysis, both descriptive and analytical logistic regression were conducted. In the descriptive analysis, all the socio-economic, demographic, and health-related variables were summarized by using frequencies (N) and percentages (%). For continuous variables, age and PHQ-9 score, mean (M) and standard deviation (SD) were calculated. All the variables that were statistically significant in the bivariate chi-square test, to detect primary association between depression and other factors, were subsequently assessed for multicollinearity using the Variance Inflation Factor (VIF) and tolerance statistics. The VIF values ranged from 1.07 to 1.93, and tolerance values ranged from 0.52 to 0.94, indicating no potential evidence of multicollinearity. Finally, the logistic regression analysis was performed. In the logistic regression model, both crude and adjusted odds were calculated to detect the association between dependent and independent variables. The regression results were reported as Crude Odds Ratios (COR) and Adjusted Odds Ratios (AOR) with 95% Confidence Intervals (CI). A two-tailed *p*-value < 0.05 was considered statistically significant. All statistical analyses were conducted using SAS version 9.4 statistical software.

### Ethical permission and consent

Before data collection, participants were verbally informed about the purpose, objectives, and potential benefits of the study. They were assured that their personal information would remain confidential and would not be disclosed. Oral consent has been taken from each participant before data collection. Ethical approval for this research was obtained from the Institutional Ethics Committee (IEC) of Sylhet Agricultural University, Bangladesh (Approval No. ARP2024030, dated July 25, 2024).

## Results

### Demographic statistics

Data were collected from a total of n = 687 participants, with an average age of 30.62 ± 11.18 years (M ± SD). Participants were drawn from all eight administrative divisions of Bangladesh, with the largest proportion from Dhaka (42.4%), followed by Sylhet (29.4%) and Mymensingh (11.5%). The majority (70.74%) were young adults aged 18–34 years, and nearly half were married. 94.0% participants belong to the Islamic religion, and the rest of them were from Hindu religious practices. In terms of occupation, 53.43% were students and 37.7% were housewives. Most participants (96.51%) had at least a secondary school education, while only 3.49% were illiterate. Two-thirds (66.23%) resided in urban areas, and social media was the most common source of political unrest-related information (39.3%). Sources of information about political unrest varied. Social media was the primary channel (39.3%), followed by family, friends, newspapers, and TV combined (33.3%), and television alone (12.1%). A smaller proportion relied on all available sources (13.5%) or direct witnessing (1.8%) (Table 1).

**Table 1:** Descriptive analysis of socio-economic, demographic, and individual-related variables (N = 14)

Variable names	Total N (%)	Depression		Chi-square	*p*-value
		No N (%)	Yes N (%)		
*Religion*					
Islam	646 (94.03)	289 (42.07)	357 (51.97)	0.65	0.42
Hinduism	41 (5.97)	21 (3.06)	20 (2.91)		
*Age groups*					
Young (18–34 years)	486 (70.74)	212 (30.86)	274 (39.88)	2.05	0.36
Middle-aged (35–54 years)	168 (24.45)	80 (11.64)	88 (12.81)		
Old (55–75)	33 (4.81)	18 (2.62)	15 (2.18)		
*Marital status*					
Married	342 (49.78)	164 (23.87)	178 (25.91)	2.28	0.32
Unmarried	337 (49.06)	143 (20.82)	194 (28.24)		
Divorced or widowed	8 (1.16)	3 (0.44)	5 (0.73)		
*Education status*					
Illiterate	24 (3.49)	5 (0.73)	19 (2.77)	21.78	0.0002
Primary school (Grade 1–5)	60 (8.73)	18 (2.62)	42 (6.11)		
Secondary school (Grade 6–10)	87 (12.66)	46 (6.70)	41 (5.97)		
Higher secondary (Grade 11–12)	141 (20.52)	80 (11.64)	61 (8.88)		
University degree (Bachelor & above)	375 (54.6)	161 (23.44)	214 (31.15)		
*Occupational status*					
Unemployed	5 (0.73)	3 (0.44)	2 (0.29)	0.95	0.81
Employed	56 (8.14)	25 (3.64)	31 (4.51)		
Homemaker	259 (37.7)	121 (17.64)	138 (20.12)		
Student	367 (53.43)	161 (23.44)	206 (29.99)		
*Living area during political unrest*					
Rural	232 (33.77)	118 (17.18)	114 (16.59)	4.66	0.031
Urban	455 (66.23)	192 (27.95)	263 (38.28)		
*Living arrangement during unrest*					
Non-solitary	661 (96.22)	301 (43.81)	360 (52.41)	1.21	0.27
Solitary (Alone)	26 (3.78)	9 (1.31)	17 (2.47)		
*Obtained real-time unrest information*					
No	193 (28.10)	102 (14.85)	91(13.25)	6.47	0.011
Yes	494 (71.90)	208 (30.28)	286 (41.62)		
*Family member affected*					
No	547 (79.62)	264 (38.43)	283(41.19)	10.68	0.001
Yes	140 (20.38)	46 (6.70)	94 (13.68)		
*Received any sad news*					
No	531 (77.29)	245 (35.66)	286(41.63)	0.97	0.32
Yes	156 (22.71)	65 (9.46)	91 (13.25)		
*Sources of gathering information on political unrest (info source)*					
All sources	93 (13.54)	45 (6.55)	48 (6.99)	27.96	< 0.0001
Directly witnessed	12 (1.75)	3 (0.44)	9 (1.31)		
*F&F, NP, SM & TV	229 (33.33)	131 (19.07)	98 (14.26)		
Social media	270 (39.3)	107 (15.57)	163 (23.73)		
Television (TV)	83 (12.08)	24 (3.49)	59 (8.59)		
*Took a sleeping pill during the political unrest*					
No	625 (90.98)	291 (42.36)	334 (48.62)	5.77	0.016
Yes	62 (9.02)	19 (2.77)	43 (6.26)		
*Took other medications during political unrest*					
No	504 (73.36)	226 (32.90)	278 (40.47)	0.06	0.81
Yes	183 (26.64)	84 (12.23)	99 (14.41)		
*The participant belonged to which of the eight divisions of Bangladesh*					
Barishal	22 (3.20)	7 (1.02)	15 (2.18)	15.41	0.031
Chittagong	33 (4.80)	14 (2.04)	19 (2.77)		
Dhaka	290 (42.21)	112 (16.30)	178 (25.91)		
Khulna	9 (1.31)	3 (0.44)	6 (0.87)		
Mymensingh	79 (11.50)	45 (6.55)	34 (4.95)		
Rajshahi	41 (5.97)	20 (2.91)	21 (3.06)		
Rangpur	11 (1.60)	6 (0.87)	5 (0.73)		
Sylhet	202 (29.40)	103 (14.99)	99 (14.41)		

*F&F, NP, SM, and TV: Friends and Family, Newspaper, Social Media, and Television, respectively

### Distribution of depression

Overall, 54% of participants were found to be depressed, with an average PHQ-9 score of 15.53 ± 4.44 (M ± SD). Depression symptoms were more frequently found among younger women (39.9%) compared to middle-aged (12.8%) and older groups (2.2%). Regarding marital status, nearly half of the participants were married (49.8%), and a similar proportion were unmarried (49.1%), with only a small proportion divorced or widowed (1.16%). Depression was most prevalent among unmarried women (28.2%) compared to married women (25.9%). However, depression symptoms were particularly frequent among those with a university degree (31.2%). In terms of occupational status, most of the women in the study population belonged to the student and homemaker categories, whereas employed women (8.14%) and unemployed women (0.73%) were the least represented groups. Depression was reported more among students (30.0%) and homemakers (20.1%). Similarly, urban residents reported higher depression prevalence (38.3%) than rural participants (16.6%). Also, depression symptoms were most common among those informed through social media (23.7%). Regarding health behaviors, 9.0% of participants reported using sleeping pills during the unrest, and 26.6% took medications for other health issues (e.g., fever, pain, vomiting, etc.). Depression was higher among those who reported any kind of medication use during the study (Table 1).

### Self-reported health outcomes

Table 2 shows the prevalence of self-reported depression-related symptoms among participants and their association with depression status using a Chi-square test. Nervousness (91.70%), loss of pleasure (86.03%), and anxiety (85.15%) were the most frequently reported symptoms. Participants reporting these symptoms had significantly higher proportions of depression (nervousness 52.84%, loss of pleasure 52.11%, anxiety 51.24%), all with *p* < 0.05. Other symptoms, including insomnia, loss of energy, frequent urination, and suffocation, were also significantly associated with depression (*p* < 0.05), indicating the existence of an association between self-reported psychosomatic symptoms and depressive status (Table 2).

**Table 2:** Descriptive analysis of health outcome-related variables (N = 7)

Variable names	Frequency N (%)	Depression status		Chi-square	*P*-value
		No N (%)	Yes N (%)		
*Anxiety*					
No	102 (14.85)	77 (11.21)	25 (3.64)	44.61	< 0.0001
Yes	585 (85.15)	233 (33.92)	352 (51.24)		
*Frequent urination*					
No	563 (81.95)	277 (40.32)	286 (41.63)	20.94	< 0.0001
Yes	124 (18.05)	33 (4.80)	91 (13.25)		
*Insomnia*					
No	230 (33.48)	147 (21.40)	83 (12.08)	49.29	< 0.0001
Yes	457 (66.52)	163 (23.73)	294 (42.79)		
*Loss of energy*					
No	349 (50.80)	202 (29.40)	147 (21.40)	46.61	< 0.0001
Yes	338 (49.20)	108 (15.72)	230 (33.48)		
*Loss of pleasure*					
No	96 (13.97)	77 (11.21)	19 (2.77)	55.47	< 0.0001
Yes	591 (86.03)	233 (33.92)	358 (52.11)		
*Nervousness*					
No	57 (8.30)	43 (6.26)	14 (2.04)	23.07	< 0.0001
Yes	630 (91.70)	267 (38.86)	363 (52.84)		
*Suffocation*					
No	273 (39.74)	176 (25.62)	97 (14.12)	68.47	< 0.0001
Yes	414 (60.26)	134 (19.51)	280 (40.76)		

### Logistic regression

The binomial logistic regression has been performed for the variables that were statistically significant in Tables 1 and 2. A total of fourteen statistically significant variables were transferred in the logistic regression analysis. In Table 1 there were seven variables that was statistically significant, such as education, living area, real-time monitoring, family members affected, source of information gathering, taking sleeping pills, and belonging to eight divisions of the country. On the other hand, all the seven self-reported symptoms were statistically significant, and all these variables were included in the logistic regression model to calculate the crude and adjusted odds. A *p*-value < 0.05 was considered statistically significant. The crude odds ratio (COR) and adjusted odds ratio (AOR) were depicted to describe the level of depression among the variables.

The binomial logistic regression analysis identified several factors significantly associated with depression among adult women following the July 2024 revolution. In the adjusted model, women whose family members were affected by the unrest had significantly higher odds of depression compared to those not affected (AOR = 2.15, 95% CI 1.35–3.40, *p* < 0.05). Educational attainment showed a protective effect, as participants with secondary (AOR = 0.20, 95% CI 0.06–0.67, *p* < 0.05), higher secondary (AOR = 0.14, 95% CI 0.04–0.48, *p* < 0.05), and university education (AOR = 0.19, 95% CI 0.06–0.62, *p* < 0.05) had significantly lower odds of depression compared to illiterate women. Regarding information sources, those who accessed unrest-related information from all sources (AOR = 0.40, 95% CI 0.23–0.69, *p* < 0.05) and from combined sources including friends and family, newspapers, social media, and television (AOR = 0.40, 95% CI 0.25–0.63, *p* < 0.05) were less likely to experience depression compared to those relying solely on social media. Additionally, psychological symptoms such as suffocation (AOR = 2.10, 95% CI 1.33–3.32, *p* < 0.05), loss of pleasure (AOR = 3.59, 95% CI 1.91–6.74, *p* < 0.05), and loss of energy (AOR = 1.70, 95% CI 1.13–2.55, *p* < 0.05) were significantly associated with higher odds of depression. However, variables including living area, receipt of real-time unrest information, sleeping pill use, primary education, direct exposure to unrest, regional divisions, anxiety, nervousness, insomnia, and increased urination were not statistically significant in the adjusted model (*p* ≥ 0.05). Overall, the findings suggest that both socio-demographic and psychological factors play a crucial role in influencing depression among women in this context (Table 3).

**Table 3:** Binomial logistic regression analysis

Variable names	Crude model			Adjusted model			*p*-value
	Est	COR^#^	95% CI	Est	AOR^@^	95% CI	
*Family members affected*							
No	1	1	1	1	1	1	–
Yes	0.65	1.91	(1.30, 2.82)	0.78	2.15	(1.35, 3.40)	0.001*
*Living area during political unrest*							
Rural	1	1	1	1	1	1	–
Urban	0.35	1.42	(1.03, 1.95)	− 0.07	1.01	(0.67, 1.52)	0.95
*Received real-time unrest information*							
No	1	1	1	1	1	1	–
Yes	0.43	1.54	(1.10, 2.15)	0.21	1.31	(0.87, 1.95)	0.19
*Used a sleeping pill during the political unrest*							
No	1	1	1	1	1	1	–
Yes	0.68	1.97	(1.12, 3.46)	0.39	1.39	(0.71, 2.73)	0.33
*Educational level*							
Illiterate	1	1	1	1	1	1	–
Primary	0.48	0.61	(0.20, 1.90)	− 0.38	0.63	(0.18, 2.28)	0.48
Secondary	− 0.71	0.24	(0.08, 0.69)	− 1.58	0.20	(0.06, 0.67)	0.01*
Higher Secondary	0.68	0.20	(0.07, 0.57)	− 1.93	0.14	(0.04, 0.48)	0.002*
University	− 0.36	0.35	(0.13, 0.96)	− 1.65	0.19	(0.06, 0.62)	0.006*
*Source of unrest information*							
Social media	1	1	1	1	1	1	–
All sources	− 0.36	0.70	(0.44, 1.13)	− 0.89	0.40	(0.23, 0.69)	0.001*
Directly witnessed	0.68	1.97	(0.52, 7.44)	0.40	1.74	(0.42, 7.26)	0.45
F&F, NP, SM & TV**	− 0.71	0.49	(0.34, 0.70)	− 0.97	0.40	(0.25, 0.63)	< 0.0001*
Television (TV)	0.48	1.61	(0.95, 2.57)	− 0.29	0.78	(0.42, 1.46)	0.43
*Divisions of participants*							
Rangpur	1	1	1	1	1	1	–
Chittagong	− 0.46	0.63	(0.20, 1.97)	− 0.60	0.55	(0.15, 1.96)	0.36
Dhaka	− 0.30	0.74	(0.29, 1.88)	− 0.07	0.94	(0.33, 2.63)	0.89
Khulna	− 0.07	0.93	(0.18, 4.87)	0.27	1.31	(0.21, 8.00)	0.77
Mymensingh	− 1.04	0.35	(0.13, 0.96)	− 0.93	0.40	(0.13, 1.25)	0.11
Rajshahi	− 0.71	0.49	(0.17, 1.45)	− 0.82	0.44	(0.13, 1.47)	0.18
Sylhet	− 0.94	0.39	(0.09, 1.72)	− 0.83	0.44	(0.09, 2.24)	0.32
Barishal	− 0.80	0.45	(0.18, 1.15)	− 0.57	0.57	(0.19, 1.65)	0.30
*Anxiety*							
No	1	1	1	1	1	1	–
Yes	1.54	4.65	(2.88, 7.52)	0.27	1.49	(0.77, 2.88)	0.24
*Nervousness*							
No		1	1	1	1	1	–
Yes	1.43	4.18	(2.24, 7.79)	0.51	1.56	(0.73, 3.32)	0.25
*Insomnia*							
No	1	1	1	1	1	1	–
Yes	1.16	3.19	(2.30, 4.44)	− 0.16	0.85	(0.52, 1.39)	0.51
*Suffocation*							
No	1	1	1	1	1	1	–
Yes	1.33	3.79	(2.75, 5.23)	0.79	2.10	(1.33, 3.32)	0.001*
*Loss of pleasure*							
No	1	1	1	1	1	1	–
Yes	1.83	6.23	(3.67, 10.56)	1.16	3.59	(1.91, 6.74)	< 0.0001*
*Loss of energy*							
No	1	1	1	1	1	1	–
Yes	1.07	2.93	(2.14, 4.00)	0.66	1.70	(1.13, 2.55)	0.01*
*Increased urination*							
No	1	1	1	1	1	1	–
Yes	0.98	2.67	(1.74, 4.11)	0.27	1.28	(0.77, 2.10)	0.34

^#^COR = Crude Odds, ^@^AOR = Adjusted Odds, *Statistically Significant at *P-*value < 0.05, 1 = Reference Category, **F&F, NP, SM, and TV: Friends and Family, Newspaper, Social Media, and Television, respectively

## Discussion

The findings of this study revealed a strikingly high prevalence 54.87% of depression among Bangladeshi women aged 18–34 years during the July 2024 revolution, underscoring the profound psychological toll of political unrest. Sayeed et al. (2023) also reported they also found up to 30% adult women of Bangladesh were in a depressed condition during the nationwide cross-sectional survey [34]. While the immediate violence of the movement marked by killings, injuries, and gender-based assaults was devastating, the aftermath has continued to perpetuate mental distress. In this study, the prevalence of nervousness, loss of pleasure, and anxiety were 91.70%, 86.03%, and 85.15%, respectively. Ahmed et al. (2025) revealed that 19.5% of the adult women in Bangladesh were anxious condition during a national survey [35]. In the modern era, participants receive most of the updated news from social media. Sometimes, social media conveys sad or negative news to users and creates depression. In this study, 23.73% of the participants show depressive symptoms. Das et al. (2025) revealed that 31.6% of social media users in Bangladesh were in a depressed condition [36]. When continuous political unrest-related news is gathered by the participants, it produces nervousness and ultimately leads to a depressed condition. This study found that among the self-reported nervous participants, 52.84% had depression. However, if the family members were directly affected during the political unrest, then those participants had 2.15 times higher odds of depression. No papers have been published regarding the family members affected and the depression level. Pearson et al. (2025) conducted research regarding the same issue during the political unrest in Myanmar, and they found that adult women had 97% of higher odds of having depression during and after the political unrest [37]. The self-reported symptoms of feeling suffocation, loss of pleasure, and energy loss had the 10%, 59%, and 70% higher odds of association with depression. Hirota et al. (2021) conducted research, and they found that among the loss of pleasure, participants had 32% higher odds of depression [38]. Hou et al. [39] found the same depression symptoms among the female citizens in Hong Kong during a civil unrest. Following the transition to an interim government, citizens anticipated justice, stability, and restoration of fundamental rights. Instead, widespread reports of extrajudicial killings, harassment, robberies, weak legal enforcement, and an absence of accountability have deepened frustration [40]. The expectation of meaningful reform was replaced by disillusionment, reinforcing cycles of recurrent depression, particularly among women who remain socially and economically vulnerable.

Frequent protests by various occupational and political groups contribute to ongoing instability, leaving little room for societal healing. Women, in particular, bear the compounded burden of trauma from witnessing violence, enduring uncertainty about safety, and struggling with disrupted livelihoods [41]. Unlike Sri Lanka, which managed partial recovery after its crisis, or Nepal’s recent 2025 (September, 2025) reforms showing hopeful trajectories [42], Bangladesh has failed to translate the sacrifices of the July revolution into tangible improvements. This unresolved turmoil fosters persistent psychological distress, eroding resilience within communities. Our findings highlight how political unrest extends beyond physical casualties to generate long-term mental health crises. Without strong governance, justice, and psychosocial support systems, depression and trauma will remain pervasive. Additionally, participants residing in geographical areas at the origin of political unrest were more likely to be depressed. Jayasuriya et al. [43] revealed that in Sri Lanka, the women who were living in the unrest-prone area were more depressed than those in the other regions.

This study revealed that over half of Bangladeshi women experienced depression during the July 2024 political revolution, with young, unmarried, and urban women most affected. Many Bangladeshi women experience depression silently due to deep-rooted cultural norms, religious obligations, and social expectations that discourage open discussion of emotional distress [44]. Feelings of shyness, fear of stigma, and concern about family reputation often led women to internalize psychological suffering rather than seek support [41]. This cultural context may have influenced self-reporting patterns and partially explains the high burden of depressive symptoms observed in this study.

These findings are consistent with Pearson et al. [44] who reported similar vulnerability among young women in Myanmar, and with Anjum et al. [45] who found higher depression among urban women in Bangladesh. Social media use was also linked to depressive symptoms, aligning with Lin et al. [46]. Although our study found no protective effect of daily physical health medication, Engström et al. [47] reported reductions in depression with mental health-related treatment. Self-reported symptoms paralleled those identified by Hou et al. [39] during Hong Kong unrest. Overall, our findings reinforce regional and global evidence that political unrest disproportionately harms women’s mental health [39].

### Limitations

This study has several limitations that should be acknowledged. First, the cross-sectional design restricts causal inference and does not allow assessment of temporal or long-term mental health effects following the July 2024 revolution. Second, the use of non-probability convenience sampling may have introduced selection bias, with potential overrepresentation of urban and educated women, limiting generalizability. Third, depressive symptoms and related exposures were self-reported, which may be influenced by recall bias or social desirability bias, particularly given mental health stigma in Bangladesh. Clinical validation of depressive symptoms was not feasible due to ethical, logistical, and security constraints during the post-revolution period. Additionally, social media exposure was assessed in aggregate, without platform-specific or content-level differentiation, which limits the nuanced interpretation of its mental health impact. In spite of that, this study has a few strengths, such as using the PHQ-9 to ensure the high measurement validity of depression. Secondly, this is a contemporary snapshot of the depression during the political unrest and ensures the original research findings.

### Future directions and implications

Future research should employ longitudinal and stratified sampling designs to examine delayed and long-term mental health consequences of political unrest and to improve population representativeness. More detailed assessments of socio-economic stressors, employment insecurity, academic pressure, and platform-specific social media exposure are warranted. Qualitative and mixed-methods studies could help elucidate trauma pathways, coping behaviors, and the role of self-medication practices such as sleeping pill use. Given the potential consequences of untreated depressive symptoms, including impaired functioning and reduced quality of life, future work should also focus on developing culturally sensitive, community-based mental health interventions and policy-relevant support strategies for women affected by socio-political crises.

## Conclusions

This study highlights the significant psychological burden experienced by women during the July 2024 political revolution in Bangladesh, with more than half reporting depressive symptoms. Young women, those unmarried, and those living in urban areas were particularly vulnerable, underscoring the importance of considering sociodemographic factors when addressing mental health during periods of political instability. Social media exposure, residence in unrest-origin areas, and the use of sleeping pills further exacerbated the risk of depression, while women on regular medication for physical conditions reported relatively lower symptoms. These findings emphasize the urgent need for targeted interventions, including mental health support systems, awareness programs, and accessible counseling services, to mitigate the long-term consequences of political unrest on women’s mental health.

## Data Availability

The data used in this study are available from the corresponding author upon reasonable request.

## References

[1] OHCHR. Human rights violations and abuses related to the protests of July and August 2024 in Bangladesh. 2024. https://www.ohchr.org/en/documents/country-reports/ohchr-fact-finding-report-human-rights-violations-and-abuses-related

[2] RTV. Death toll in July revolution reaches 1423 over 22,000 injured. 2024. https://rtvonline.com/english/bangladesh/16684

[3] European Union. Union agency for Asylum E. COI report–Bangladesh: country focus. 2025. 10.2847/0041862

[4] United Nations Bangladesh. Bangladesh: UN report finds brutal, systematic repression of protests, calls for justice for serious rights violations. 2025. https://bangladesh.un.org/en/289108-bangladesh-un-report-finds-brutal-systematic-repression-protests-calls-justice-serious

[5] The Dailly Star. July 2024 mass protests Bangladesh injuries. Shot, July heroes battle a harsh new reality. 2024. https://tds-images.thedailystar.net/news/bangladesh/news/shot-july-heroes-battle-harsh-new-reality-3767041

[6] The Daily Star. Female martyrs of July uprising Bangladesh 2024; tales of 10 women killed in July uprising. 2024. https://www.thedailystar.net/news/bangladesh/news/tales-10-women-killed-july-uprising-3842246

[7] Rasgon N, Myoraku A. Special report: advancing mood disorder care—from parsimony to precision. Psychiatry News. 2023. 10.1176/APPI.PN.2023.10.10.1.

[8] WHO. Depression. 2017. https://www.who.int/health-topics/depression

[9] Ni MY, Kim Y, McDowell I, Wong S, Qiu H, Wong IOL, et al. Mental health during and after protests, riots and revolutions: a systematic review. Aust N Z J Psychiatry. 2020;54:232–43. 10.1177/0004867419899165. 31989834 10.1177/0004867419899165

[10] Alon N, Macrynikola N, Jester DJ, Keshavan M, Reynolds CF, Saxena S, et al. Social determinants of mental health in major depressive disorder: umbrella review of 26 meta-analyses and systematic reviews. Psychiatry Res. 2024;335:115854. 10.1016/J.PSYCHRES.2024.115854. 38554496 10.1016/j.psychres.2024.115854

[11] Kibris A, Goodwin R. The long-term effects of war exposure on psychological health: An experimental study with Turkish conscript veterans. Soc Sci Med. 2024;340:116453. 10.1016/J.SOCSCIMED.2023.116453. 38061221 10.1016/j.socscimed.2023.116453

[12] Steel Z, Chey T, Silove D, Marnane C, Bryant RA, Van Ommeren M. Association of torture and other potentially traumatic events with mental health outcomes among populations exposed to mass conflict and displacement: a systematic review and meta-analysis. JAMA. 2009;302:537–49. 10.1001/JAMA.2009.1132. 19654388 10.1001/jama.2009.1132

[13] Lim ICZY, Tam WWS, Chudzicka-Czupała A, McIntyre RS, Teopiz KM, Ho RC, et al. Prevalence of depression, anxiety and post-traumatic stress in war- and conflict-afflicted areas: A meta-analysis. Front Psychiatry. 2022. 10.3389/FPSYT.2022.978703. 36186881 10.3389/fpsyt.2022.978703 PMC9524230

[14] Shoib S, Maqbool Dar M, Bashir H, Qayoom G, Arif T. Psychiatric morbidity and the socio-demographic determinants of patients attempting suicide in Kashmir Valley: A cross-sectional study. Int J Health Sci Res. 2012;2:45.

[15] Mollica RF, McInnes K, Poole C, Tor S. Dose-effect relationships of trauma to symptoms of depression and post-traumatic stress disorder among Cambodian survivors of mass violence. Br J Psychiatry. 1998;173(DEC.):482–8. 10.1192/BJP.173.6.482. 9926076 10.1192/bjp.173.6.482

[16] Sri Lanka Brief. The Psychological Impact of Political Violence in Sri Lanka–Dr Ruwan M Jayatunge M.D. Sri Lanka Brief. 2025. https://srilankabrief.org/the-psychological-impact-of-political-violence-in-sri-lanka-dr-ruwan-m-jayatunge-m-d/

[17] Husain F, Anderson M, Lopes Cardozo B, Becknell K, Blanton C, Araki D, et al. Prevalence of war-related mental health conditions and association with displacement status in Postwar Jaffna District, Sri Lanka. JAMA. 2011;306(5):522–31. 10.1001/JAMA.2011.1052. 21813430 10.1001/jama.2011.1052

[18] Polyvianaia M, Yachnik Y, Fegert JM, Sitarski E, Stepanova N, Pinchuk I. Mental health of university students twenty months after the beginning of the full-scale Russian-Ukrainian war. BMC Psychiatry. 2025. 10.1186/S12888-025-06654-1. 40075321 10.1186/s12888-025-06654-1 PMC11905627

[19] Blackmore R, Boyle JA, Fazel M, Ranasinha S, Gray KM, Fitzgerald G, et al. The prevalence of mental illness in refugees and asylum seekers: a systematic review and meta-analysis. PLoS Med. 2020. 10.1371/JOURNAL.PMED.1003337. 32956381 10.1371/journal.pmed.1003337 PMC7505461

[20] Amiri S, Khan MAB. Trends in prevalence and burden of depressive disorders in Iran at national and subnational levels: estimates based on sex and age groups. Gen Psychiatry. 2025. 10.1136/GPSYCH-2024-102016. 10.1136/gpsych-2024-102016 PMC12182112 40547356

[21] Amsalem D, Haim-Nachum S, Lazarov A, Levi-Belz Y, Markowitz JC, Bergman M, et al. The effects of war-related experiences on mental health symptoms of individuals living in conflict zones: a longitudinal study. Sci Rep. 2025;15(1):1–9. 10.1038/S41598-024-84410-3;SUBJMETA. 39762464 10.1038/s41598-024-84410-3 PMC11704351

[22] Gebreyesus A, Niguse AT, Shishay F, Mamo L, Gebremedhin T, Tsegay K, et al. Prevalence of depression and associated factors among community hosted internally displaced people of Tigray; during war and siege. BMC Psychiatry. 2024;24(1):1–9. 10.1186/S12888-023-05333-3/TABLES/4. 38166772 10.1186/s12888-023-05333-3 PMC10763281

[23] WHO. Investing in treatment for depression and anxiety leads to fourfold return. 2016. https://www.who.int/news/item/13-04-2016-investing-in-treatment-for-depression-and-anxiety-leads-to-fourfold-return

[24] Khan R, Akter F, Rahman M, Amin N, Rahman M, Winch PJ. Socio-demographic factors associated with mental health disorders among rural women in Mymensingh, Bangladesh. Front Psychiatry. 2025. 10.3389/FPSYT.2025.1446473. 40041695 10.3389/fpsyt.2025.1446473 PMC11876128

[25] Ahsan MS, Sarkar AA, Khan MA-A, Ahmed T, Islam MR, Akter S, et al. Mental health consequences of the July revolution in Bangladesh: a study on depression and post-traumatic stress disorder among survivors of violence and persecution. Cureus. 2025;17(5):358. 10.7759/CUREUS.84358. 10.7759/cureus.84358 PMC12173962 40535373

[26] Jaim J. Problems of political unrest: women in small businesses in Bangladesh. N Engl J Entrep. 2022;25(1):4. 10.1108/NEJE-01-2021-0004.

[27] Perkins JD, Ajeeb M, Fadel L, Saleh G. Mental health in Syrian children with a focus on post-traumatic stress: a cross-sectional study from Syrian schools. Soc Psychiatry Psychiatry Epidemiol. 2018;53(11):1231–9. 10.1007/S00127-018-1573-3. 10.1007/s00127-018-1573-3 PMC6208941 30083987

[28] Kalaitzaki A, Goodwin R, Kurapov A, Vintila M, Lazarescu G, Lytvyn S, et al. The mental health toll of the Russian-Ukraine war across 11 countries: Cross-sectional data on war-related stressors, PTSD and CPTSD symptoms. Psychiatry Res. 2024;342:116248. 10.1016/J.PSYCHRES.2024.116248. 39476454 10.1016/j.psychres.2024.116248

[29] Anbesaw T, Kassa MA, Yimam W, Kassaw AB, Belete M, Abera A, et al. Factors associated with depression among war-affected population in Northeast, Ethiopia. BMC Psychiatry. 2024;24(1):376. 10.1186/S12888-024-05812-1. 38773453 10.1186/s12888-024-05812-1 PMC11106904

[30] Bener A, Al-Kazaz M, Ftouni D, Al-Harthy M, Dafeeah EE. Diagnostic overlap of depressive, anxiety, stress and somatoform disorders in primary care. Asia Pac Psychiatry. 2013;5(1):29. 10.1111/J.1758-5872.2012.00215.X. 10.1111/j.1758-5872.2012.00215.x 23857793

[31] Löwe B, Spitzer RL, Williams JBW, Mussell M, Schellberg D, Kroenke K. Depression, anxiety and somatization in primary care: syndrome overlap and functional impairment. Gen Hosp Psychiatry. 2008;30(3):191–9. 10.1016/J.GENHOSPPSYCH.2008.01.001. 18433651 10.1016/j.genhosppsych.2008.01.001

[32] Penninx BWJH, Milaneschi Y, Lamers F, Vogelzangs N. Understanding the somatic consequences of depression: biological mechanisms and the role of depression symptom profile. BMC Med. 2013;11(1):1–14. 10.1186/1741-7015-11-129/TABLES/3. 23672628 10.1186/1741-7015-11-129 PMC3661358

[33] Vaccarino AL, Sills TL, Evans KR, Kalali AH. Prevalence and association of somatic symptoms in patients with major depressive disorder. J Affect Disord. 2008;110(3):270–6. 10.1016/j.jad.2008.01.009. 18280580 10.1016/j.jad.2008.01.009

[34] Sayeed A, Rahman MH, Hassan MN, deSteiguer A, Kundu S, Meem AE, et al. Prevalence and associated factors of depression among Bangladeshi university students: a cross-sectional study. J Am Coll Health. 2023;71(5):1381–6. 10.1080/07448481.2021.1944168. 34469258 10.1080/07448481.2021.1944168

[35] Ahmed E, Methun MIH, Siam MR. Determinants of depression and anxiety among reproductive-aged women in Bangladesh: insights from national survey. J Affect Disord. 2025;390:119771. 10.1016/J.JAD.2025.119771. 40582596 10.1016/j.jad.2025.119771

[36] Das S, Zubayer AA, Snigdha MF, Uddin MF, Afridi MK, Kanak KFJ, et al. Social media use time and mental health of young adults: a cross-sectional study in Bangladesh. Health Sci Rep. 2025;8(7):e71117. 10.1002/HSR2.71117. 40709074 10.1002/hsr2.71117 PMC12286897

[37] Pearson I, Chase E, Van Kim C, San NM, Ja H, Hlaing ZM, et al. Conflict exposure and mental health: a survey of adolescent girls and young women in Myanmar post the 2021 coup d’état. Confl Health. 2025;19(1):29. 10.1186/S13031-025-00668-Y. 40380322 10.1186/s13031-025-00668-y PMC12082879

[38] Hirota T, McElroy E, So R. Network analysis of internet addiction symptoms among a clinical sample of Japanese adolescents with autism spectrum disorder. J Autism Dev Disord. 2021;51(8):2764–72. 10.1007/S10803-020-04714-X. 33040268 10.1007/s10803-020-04714-x

[39] Hou WK, Li TW, Liang L, Liu H, Ettman CK, Hobfoll SE, et al. Trends of depression and anxiety during massive civil unrest and COVID-19 in Hong Kong, 2019–2020. J Psychiatry Res. 2022;145:77–84. 10.1016/j.jpsychires.2021.11.037. 10.1016/j.jpsychires.2021.11.037 PMC8636149 34875462

[40] Press Express. Bangladesh sees surge in extrajudicial killings, custodial deaths under interim government. 2025. https://pressxpress.org/2025/08/30/bangladesh-sees-surge-in-extrajudicial-killings-custodial-deaths-under-interim-government/

[41] United Nations in Bangladesh. Gender analysis Bangladesh (July–December 2024) impact of the civil unrest on women and Marginalised groups. 2024. https://bangladesh.un.org/en/290655-gender-analysis-bangladesh-july-%E2%80%93-december-2024-impact-civil-unrest-women-and-marginalised

[42] Hindustan Times. Nepal PM vows reforms as new govt takes shape. 2025. https://www.msn.com/en-in/news/India/nepal-pm-vows-reforms-as-new-govt-takes-shape/ar-AA1MxFK5

[43] Jayasuriya D, Jayasuriya R, Tay AK, Silove D. Associations of mental distress with residency in conflict zones, ethnic minority status, and potentially modifiable social factors following conflict in Sri Lanka: a nationwide cross-sectional study. Lancet Psychiatry. 2016;3(2):145–53. 10.1016/S2215-0366(15)00437-X. 26796018 10.1016/S2215-0366(15)00437-X

[44] Pearson I, Chase E, Van Kim C, San NM, Ja H, Hlaing ZM, et al. Conflict exposure and mental health: a survey of adolescent girls and young women in Myanmar post the 2021 coup d’état. Confl Health. 2025;19(1):1–12. 10.1186/S13031-025-00668-Y/TABLES/7. 40380322 10.1186/s13031-025-00668-y PMC12082879

[45] Anjum A, Hossain S, Sikder T, Uddin ME, Rahim DA. Investigating the prevalence of and factors associated with depressive symptoms among urban and semi-urban school adolescents in Bangladesh: a pilot study. Int Health. 2022;14(4):354–62. 10.1093/INTHEALTH/IHZ092. 31693088 10.1093/inthealth/ihz092 PMC10575608

[46] Lin LY, Sidani JE, Shensa A, Radovic A, Miller E, Colditz JB, et al. Association between social media use and depression among U.S. young adults. Depress Anxiety. 2016;33(4):323. 10.1002/DA.22466. 26783723 10.1002/da.22466 PMC4853817

[47] Engström I, Hansson L, Ali L, Berg J, Ekstedt M, Engström S, et al. Relational continuity may give better clinical outcomes in patients with serious mental illness–a systematic review. BMC Psychiatry. 2023;23(1):1–13. 10.1186/S12888-023-05440-1/TABLES/2. 38110889 10.1186/s12888-023-05440-1 PMC10729558

